# Mobile service delivery in response to the opioid epidemic in Philadelphia

**DOI:** 10.1186/s13722-023-00427-5

**Published:** 2023-11-29

**Authors:** Rebecca E. Stewart, Hanna P. Christian, Nicholas C. Cardamone, Catherine Abrams, Caroline Drob, David S. Mandell, David Metzger, Margaret Lowenstein

**Affiliations:** 1 University of Pennsylvania Perelman School of Medicine, Philadelphia, PA USA; 2 The Health Federation of Philadelphia, Philadelphia, PA USA

**Keywords:** Opioid use disorder, Buprenorphine, Mobile treatment, HIV prevention, PWID, Low barrier treatment model

## Abstract

**Background:**

The harms of opioid use disorder (OUD) and HIV infection disproportionately impact marginalized populations, especially people experiencing homelessness and people who inject drugs (PWID). Mobile OUD service delivery models are emerging to increase access and reduce barriers to OUD care. While there is growing interest in these models, there is limited research about the services they provide, how they operate, and what barriers they face. We characterize the capacity, barriers, and sustainment of mobile OUD care services in a large city with a high incidence of OUD and HIV.

**Methods:**

From May to August 2022, we conducted semi-structured interviews with leadership from all seven mobile OUD care units (MOCU) providing a medication for OUD or other substance use disorder services in Philadelphia. We surveyed leaders about their unit’s services, staffing, operating location, funding sources, and linkages to care. Leaders were asked to describe their clinical approach, treatment process, and the barriers and facilitators to their operations. Interview recordings were coded using rapid qualitative analysis.

**Results:**

MOCUs are run by small, multidisciplinary teams, typically composed of a clinician, one or two case managers, and a peer recovery specialist or outreach worker. MOCUs provide a range of services, including medications for OUD, wound care, medical services, case management, and screening for infectious diseases. No units provide methadone, but all units provide naloxone, six write prescriptions for buprenorphine, and one unit dispenses buprenorphine. The most frequently reported barriers include practical challenges of working on a MOCU (e.g. lack of space, safety), lack of community support, and patients with substantial medical and psychosocial needs. Interviewees reported concerns about funding and specifically as it relates to providing their staff with adequate pay. The most frequently reported facilitators include positive relationships with the community, collaboration with other entities (e.g. local nonprofits, the police department, universities), and having non-clinical staff (e.g. outreach workers, peer recovery specialists) on the unit.

**Conclusions:**

MOCUs provide life-saving services and engage marginalized individuals with OUD. These findings highlight the challenges and complexities of caring for PWID and demonstrate a need to strengthen collaborations between MOCU providers and the treatment system. Policymakers should consider programmatic funding for permanent mobile OUD care services.

**Supplementary Information:**

The online version contains supplementary material available at 10.1186/s13722-023-00427-5.

## Background

Unintentional opioid-related overdose deaths continue to rise despite great efforts to provide and expand treatment for opioid use disorder (OUD) [1–3]. The surge in fatal overdoses is fueled by the rising availability of synthetic opioids (e.g. fentanyl), which have largely replaced heroin in many drug markets [4]. The fast-acting effects of fentanyl may lead to more frequent intravenous drug administration and exacerbate the risk for human immunodeficiency virus (HIV) transmission among people who inject drugs (PWID) [5, 6]. Increasing outbreaks of HIV among PWID have been documented in several states and municipalities, including Philadelphia [7–10]. Housing instability, poverty, and inadequately treated substance use disorder result in more severe complications from HIV for people with PWID, who comprise a disproportionate amount of HIV-related deaths [11–14].

An increase in the number of HIV outbreaks and opioid overdose deaths has increased the urgency of HIV prevention and OUD treatment efforts in this population, however PWID are underserved by traditional treatment programs [15–17]. Contributing factors to this treatment gap include stigma, being unhoused, and dehumanizing care in the substance use treatment system [18, 19]. In addition, many traditional treatment models impose requirements such as frequent attendance, counseling, or complete abstinence as conditions for receiving care, which may further reduce access among the most marginalized individuals most in need of care [20]. To increase access and reduce barriers to care for people who use drugs at risk for HIV, mobile care delivery models and other low threshold treatment models have emerged to deliver care in the community and connect individuals to treatment. Mobile OUD care units (MOCUs) are vehicles whose accompanying staff can provide a range of medical, behavioral, and harm reduction services, including but not limited to, HIV and viral hepatitis testing, pre-exposure prophylaxis (PrEP) and antiviral treatment, and distributing naloxone and fentanyl test strips. MOCUs offer medications for opioid use disorder (MOUD) such as buprenorphine, which can be rapidly initiated and titrated to maintenance doses without the need for daily dispensing [21–23]. Facilitated by the removal of registration requirements for mobile methadone units during the COVID-19 pandemic, MOCUs in several cities have started dispensing methadone [24, 25]. Unlike fixed treatment sites, MOCUs are able to more rapidly respond to shifting areas of drug use activity and directly serve areas where hard-to-reach groups, including PWID, live and congregate.

Research on street-based healthcare delivery and MOCUs have not kept pace with the rapid expansion of their use [26–30]. Single-case descriptions of pilot initiatives characterize much of the existing literature on mobile OUD care delivery models [31–35]. Nonetheless, the small body of empirical studies suggests that MOCUs are a promising avenue for providing access to MOUD [36–38]. One study found that providing transportation to treatment through a MOCU led to increase treatment engagement at a brick and mortar treatment facility [16]. An ongoing study is evaluating the efficacy of using mobile care delivery models that provide MOUD to people who inject opioids in five U.S. cities [39], but has not yet published results.

While mobile OUD care delivery models are intuitively appealing, there are important questions about their reach (e.g., engaging traditionally hard-to-reach populations), the effectiveness of care provided, and the feasibility and acceptability of service integration tailored towards PWID such as MOUD, HIV and viral hepatitis testing, HIV/HCV treatment, and PrEP. Gathering more evidence on MOCUs can also inform and facilitate policy and payment practices and ensure that important resources are not being diverted from other tested care models. The efficacy questions are untestable without preliminary information about current mobile OUD care service delivery including, the range of services, staff and capacity, and barriers and facilitators to optimal operation and sustainability. The current study describes the landscape of MOCUs in Philadelphia, an urban epicenter of the opioid overdose crisis, including treatment approaches, provision of services, and funding models, and identifies facilitators and barriers to their operations [3]. Based on these findings, we make recommendations to support the functioning and sustainability of mobile OUD care services in Philadelphia and beyond.

### Local policy context

Philadelphia has long-established harm reduction policies and practices to reduce harm and promote treatment among people who use drugs. Since the establishment of a state-level standing order in 2015, naloxone has been available to any Philadelphia resident without an individual prescription pharmacy [40]. Naloxone and fentanyl test strips are also widely distributed by both city agencies and harm reduction organizations [41, 42]. Although distribution of syringes is still illegal under Pennsylvania drug paraphernalia laws, a 1992 mayoral order authorized one local syringe service program (SSP), which is currently one of the largest SSPs in North America [43]. Philadelphia has also adopted a number of lower threshold care models to improve treatment access [42, 43] including telehealth models, emergency department-initiated treatment, and treatment embedded within syringe service programs [23, 44–46]. While the mayor’s office has unequivocally supported the opening of an overdose prevention site (OPS), attempts to do so have been stymied by community opposition and legal disputes. In 2023, the Pennsylvania Senate and the Philadelphia City Council voted to ban OPSs.

## Methods

From May to August 2022, we conducted semi-structured interviews with a purposive sample of leadership of MOCUs who provide MOUD and other services for PWID in Philadelphia.

Participants were recruited from a monthly learning collaborative of MOCU leaders hosted by the Health Federation of Philadelphia (CA, CD), which provides training, technical assistance, and other supports for community providers in Philadelphia. We limited study inclusion to leaders (e.g., clinical or medical director, principal investigator, executive officer) of MOCUs that provide or link to MOUD and were in operation in Philadelphia County as of February 2022. We approached seven potential participants via email; all seven agreed to participate in the study.

We designed a semi-structured interview guide to examine mobile care delivery models that provide treatment for OUD and HIV prevention (see Additional file 1). The interview guide was informed by qualitative methods in implementation science [47] and rapid qualitative analysis (RQA) frameworks [48, 49]. In line with recommendations from RQA [50], we took steps to improve the rigor of data collection. Notably, an experienced addiction medicine physician-researcher (ML) validated the interview content and conducted interviews with unit leaders. The interview guide was pilot tested with a MOCU leader and refined. Following pilot testing, we conducted an independent follow-up interview with a different leader of the same MOCU to ensure complete data collection. No repeat interviews were conducted. We sent participants an email with a description of the study and its purpose. We used a video conferencing software to conduct interviews, which were approximately 45 min in length. We asked participants to join the remote session from a private place in their homes or offices. In addition to the interviewer and interviewee, a trained research assistant took field notes during the session.

We asked interviewees about their MOCU’s structure and capacity, including staffing, case load, physical space, location, and funding mechanism. We also asked about services the MOCU provided using a list of services, including medical services, MOUDs, harm reduction services, and linkages to other supports. Finally, we asked participants about their MOCU’s clinical approach and treatment process as well as barriers and facilitators to the unit’s current and future operations and services. All interviews were recorded and transcribed verbatim by a trained research coordinator assisted by an automatic transcription software [51]. The study was determined exempt by the University of Pennsylvania IRB and followed the COREQ checklist for reporting on qualitative research (see Additional file 2) [52].

A primary goal of this study was to collect, rapidly analyze, and disseminate information back to public health officials and MOCU leaders to provide insights and facilitate collaboration between the MOCUs. A trained research coordinator (HC) collated and summarized the descriptive data from the interview and prepared tables with this information about unit structure, capacity, and services. The results of the descriptive data were returned to each MOCU, six provided confirmation and service updates and one did not respond. We employed action-oriented RQA techniques [48, 50, 53] to extract themes related to the MOCUs’ treatment approaches as well as the barriers and facilitators to their operation and implementation. During the interviews, we compiled detailed field notes using a template that corresponded with the domains on the interview guide. Following the interviews, we used transcripts to enrich in-session field noes, discuss data saturation, and extract illustrative quotations. Two authors (RS, NC) validated the summaries and condensed them into a table to help compare thematic responses across MOCUs (Additional file 3).

## Results

### ***Characteristics of MOCUs in Philadelphia*** (Table 1)

**Table 1:** Characteristics of MOCUs in Philadelphia

MOCU Name	Operating Since	Vehicle type	Primary location (s)	Typical staff on unit	Funding sources
Unit 1	2019	Custom RV	Kensington South Philadelphia	A medical professional, two peer recovery specialists, and a research coordinator	Federal research grant (part of clinical trial)
Unit 2	2019	RV	Kensington	Two medical professionals, four outreach workers, and a case manager	Parent organization
Unit 3	2021	RV	Kensington Southwest Philadelphia	Two medical professionals, two case managers, and a peer recovery specialist	City partnership, Medicaid
Unit 4	2019	RV	South Philadelphia	A medical professional case manager, one or two peer recovery specialists, and a program manager	Federal grant, Medicaid
Unit 5	2020	Shuttlebus	Kensington South Philadelphia	A medical professional, one or two case managers, and an outreach specialist	City partnership, Medicaid
Unit 6	2022	SUV	Kensington	Two medical professionals	Parent organization, Medicaid
Unit 7	2020	SUV	Kensington North Philadelphia	Two outreach specialists	City partnership

RV: Recreational vehicle, SUV: Sport Utility Vehicle

#### Physical space

Two MOCUs operate out of custom-made medical vehicles (RV) holding two exam rooms, and a bathroom. Another MOCU operates out of a retrofitted RV with lab testing equipment (e.g., centrifuge), one clinical space, and a bathroom. Two MOCUs operate out of vans–one a converted shuttle bus and the other a converted camper van–that include a clinical space but not a laboratory space. The remaining two MOCUs operate out of SUVs; these units also do outreach and travel around the city to a variety of locations to deliver harm reduction supplies.

#### Location

Most MOCUs operate in Kensington, Philadelphia, an epicenter of injection drug use and the area of the city with the highest overdose death rate in Philadelphia. Half of the MOCUs operate in other neighborhoods in the city, including South or North Philadelphia. Operating hours of the MOCUs ranged from 7 to 35 h per week, Monday through Friday. Four units stay in the same location each day of operation, and two units vary between key locations (e.g. Kensington, West Philadelphia, Southwest Philadelphia).

Six of seven MOCUs return to a specific location within a neighborhood for at least six months before choosing a new area. Among these six, two MOCUs reported serving a location for six to twelve months then using city overdose data to guide a new location to service. The two SUV MOCUs do not park but instead drive around a neighborhood to meet and transport clients as needed.

#### Staffing

MOCUs are typically staffed by three to six people. Six of the seven MOCU’s have a clinician (e.g. physician, registered nurse, nurse practitioner) on the MOCU at all times. Three of seven MOCUs employ peer recovery specialists, and three of seven MOCUs have community outreach specialists. Four MOCUs employ case managers who help clients address barriers that make traditional clinical settings difficult to access such as insurance enrollment or obtaining photo identification. One staff member, typically an outreach specialist, drives the vehicle.

#### Affiliation and funding sources

Each MOCU was supported by a larger organization. Three MOCUs are affiliated with universities located in Philadelphia and four MOCUs are part of non-profit service organizations. MOCUs are funded through federal research grants, their parent organization, city partnerships, and Medicaid reimbursement.

#### Caseload and capacity

Current caseloads ranged from 20 to over 100 individuals. MOCUs reported having anywhere from one touchpoint a week per patient for an indefinite period—to up to five times a week for the first month. Descriptions of capacity varied due to different definitions of length of stay and client status. Three units were unable to approximate a maximum capacity. One unit was unable to estimate capacity due to clients being seen at both their mobile unit and brick-and-mortar sites. One unit is a part of a randomized controlled trial study with a pre-specified capacity (52 people at maximum for a 6 month period).

### Services provided by MOCUs in Philadelphia (Table 2)

**Table 2:** Services Offered on MOCUs

Medical services	Unit 1^a^	Unit 2	Unit 3	Unit 4	Unit 5	Unit 6	Unit 7
Wound Care	O	R	O	O	O	O	R
Pregnancy testing	O	O	O	O	R	O	R
Screening for sexually transmitted infections	O	R	O	O	R	O	R
Screening for HIV and hepatitis C virus	O	R	O	O	R	O	R
**Medications**							
Naloxone distribution and education	O	O	O	O	O	O	O
Buprenorphine prescriptions	O	O	O	O	O	O	R
PrEP prescription	O	R	O	O	R	O	R
Administer Vivitrol	R	R	O	R	R	R	R
Buprenorphine dispensation	O	No	No	No	No	No	No
Administer Sublocade/Brixadi	R	R	O	R	R	R	R
Dispense methadone	R	No	R	R	R	R	R
**Harm Reduction Services**							
Fentanyl test strip distribution	R	R	R	O	O	R	R
Syringe distribution	R	R	R	R	R	R	R
**Other Supports**							
Case Management	O	R	O	O	O	O	O
Basic supplies (food, water, toiletries, clothing)	O	O	O	O	O	O	O
Transportation assistance	O	O	R	O	O	O	O
Assistance with housing	O	R	R	O	O	O	O
Assistance with obtaining insurance/benefits	O	R	O	O	O	R	O
Assistance with copays	No	O	O	O	O	O	O
Peer specialist support	O	R	O	O	O	R	O

O: onsite, R: referral

^a^This MOCU operates as an RCT, the results presented report on the investigation group/intervention arm of the study

#### Medical services

Most MOCUs provide wound care, pregnancy testing, and sexually transmitted infection testing. Four of seven MOCUs provide screening for HIV and hepatitis C virus. Every unit reported providing a form of primary care services depending on their capacity to do so, referring to higher care when necessary.

#### Medications

All MOCUs provide the overdose reversal, naloxone. Six MOCUs have a buprenorphine prescriber on the unit. Two MOCUs dispense MOUD on-site; one dispenses buprenorphine films; one provides injectable extended-release buprenorphine (i.e. Sublocade) and injectable naltrexone (i.e. Vivitrol). No MOCU provides methadone but four units clients to methadone clinics. Only one unit directly dispenses MOUD refills, but others provide a short-term “bridge” prescription. Six MOCUs prescribe PrEP for HIV.

#### Harm reduction services

Five MOCUs refer clients to other organizations to obtain fentanyl test strips and two units dispense fentanyl test strips on site. All MOCUs (and other treatment facilities) refer their clients to only local SSP to receive clean syringes.

#### Other supports

All MOCUs provide case management (e.g., procuring identification, housing, insurance), and basic supplies (e.g., food, water, toiletries, clothing). Six MOCUs offer transportation assistance through the local transportation authority or ride share services. Six MOCUs help individuals obtain housing and insurance, including providing education about co-pays. Three MOCUs provide peer support through certified peer recovery specialists employed on the unit.

### Approach to service delivery

MOCU leaders described a treatment philosophy centered around providing access and engaging underserved populations. Some leaders described their MOCU as a bridge from which they could work towards empowering their clients and building their trust in the health care system. MOCU leaders responded to several factors that disconnect individuals from the health care system, such as being unhoused, having a substance use disorder, being poorly treated in the treatment system and “*falling through the cracks.*” As one leader described,

“*I think we’re what I call…gap fillers. Anyone that’s worked in the [health] system knows, you have to accept that it’s broke before you even start working in it…there are gaps in the system, where people could get assessed, but then they never make it to the program*.”

Many MOCU leaders described their “*one stop shop*” approach of providing point-of-care services for imminent health needs such as wound care and “*bridge scripts,*” (i.e., short term prescriptions), for buprenorphine regardless of insurance status. Most of the MOCUs tried to refer individuals in community-based care. One leader clarified:

“*We’re a linkage program. We aim not to keep people; our goal is to link people to a more permanent place…we view ourselves as we're sort of a bridge.”*

### Barriers

#### Treatment system deficits

Even as MOCUs leaders sought to connect individuals to the treatment system, they identified many deficiencies in the traditional treatment system as a primary barrier to patient success. Many cited that there are too many agency requirements for care. One leader described them as, “regimented, old-school programs.” Leaders pointed out that treatment facilities that require intensive outpatient attendance to receive medication are problematic. One leader noted:

*“While that works for some people, for others, it can be a really big commitment, it prevents them from working, it prevents them from doing what they need to do to be ok.”*

Leaders zeroed in on processes such as waiting on-site for assessments and induction:

“*Right now, you increase all the services, buprenorphine access, more spots available, you can throw a lot of harm reduction supplies, and expand access to MOUD…but we’re still having an increase in overdose deaths and HIV [transmission] with drug users. The old model [the structured process: go to clinic, you need to be abstinent for 24/48 hours, then start induction]…isn’t benefitting people. People want to avoid the withdrawal, especially if they are living on the street.”*

Other treatment system barriers included the variability in whether recovery houses accepted patients on MOUD, waitlists for outpatient substance use treatment, lack of accessible mental health services, and lack of inpatient treatment beds. As one leader described:

*“We actually do have easy access to medical treatment that people need but psychiatric services are a major problem.”*

An additional difficulty was lack of access to data on the people the MOCUs served, as well as little data sharing infrastructure within the treatment system, which limits coordination and patient care*:*

*“When we finally do get people to go into the hospital, which is rare, were encountering where [the hospital] won’t give us information. You know, naming things about like HIPAA even though our folks are saying they can talk to us, or just not taking the time to involve us in the care. So it ends up being a lost opportunity that sort of further stigmatizes our folks from engaging in the healthcare system.”*

Another leader commented:

*“I’m hoping that we can all get on the same page with data… I wish there was one central place. I've done a lot of research with like other jurisdictions, in California, if you're in a hospital Monday, and you go to a different hospital Wednesday, that hospital that you go to on Wednesday, they would already know that you were in the hospital.”*

#### Patient-facing barriers

Leaders mentioned important patient-level barriers to treatment. Being unhoused and lacking a phone or other means to communicate were the most commonly mentioned. One leader described:

*“I used to think we could treat our way out of this epidemic, [however] given the co-occurring crisis in these folks lives, which I think the most important is housing, but there are lots of other issues…having reliable communications with people, [and] psychiatric services that can be delivered quickly”.*

Due to these factors, patients have difficulty attending appointments on the MOCU or for other services. Leaders also noted that their patients often feel uncomfortable in traditional facility waiting rooms:

*“You might be concerned about how you look when you present in a traditional waiting room, as well as sometimes, if you don’t have a safe place to live, leaving your stuff somewhere to come*.”

#### Community-related barriers

Several unit leaders spoke of community resistance toward PWID and the MOCU. One leader noted:

“*We've been asked to leave on many occasions, interestingly, by residents of the Southwest and by the police in Kensington and residents in Kensington*.”

Leaders described community resistance to the MOCU and a belief that the unit was attracting “undesirable” individuals to the area*.* One leader reported that a local pharmacist refused to stock buprenorphine, making it difficult for MOCU patients to fill their prescriptions. Another leader described the tension between the community and the OUD population:

*“This is a travesty. [People with OUD] need help. It's also a travesty for the people that live in the community. And how can [we help] both at the same time, knowing that it's not going to be perfect for either one, right? It's not going to be perfect for the community. It's not going to be perfect for the people we're trying to help. You hear it from the community, you guys seem like all you care about is these people, nobody cares about us.*”

Lastly, unit leaders described how increases in gun violence in the communities being serviced has led to challenges in maintaining safety and care for patients and staff. One leader likened it to delivering care, “in a war zone.”

#### Physical space and practical challenges

The MOCUs face many practical challenges. A lack of space (and lack of private space) limits MOCUs from providing the full range of services that patients would normally receive at a brick-and-mortar health center. The space also limits patient accessibility and volume:

*“[Our unit is] essentially just one room. There’s only one clinical space. We can only see one patient at a time.”*

MOCUs that lack bathrooms are unable to conduct pregnancy tests and urine screenings. Two MOCUs lack air conditioning, which makes them uncomfortable in the summer. Multiple MOCUs reported closing operations for an extended period due to vehicle servicing and repair. In addition, MOCUs lack the security benefits of buildings: several leaders reported that they are unable to keep medication on their MOCU due to concerns about theft. One leader enumerated the limits of their unit:

*“[We] can’t dispense methadone, can’t leave Narc[an] on bus, no AC, no restroom, no urine screens.”*

#### Financing and funding model

All MOCUs leaders listed lack of funding as an immediate barrier to MOCU operation and sustainability. Most MOCUs were partly or fully grant funded. Four MOCUs billed for medical services, and leaders noted that the reimbursement was inadequate to fully cover the costs for mobile services. Leaders reported that their parent organizations are small and operate on small budgets. Due to limited funding, there is a constant fear of service shutdown and an inability to hire, retain, or sufficiently compensate staff:

*“There’s always the funding issue and paying salaries for better retention: I'm a huge believer in peers and people that do that frontline work should be compensated better than they are.”*

Several leaders also report that the data collection and reporting mandated by funders is difficult to complete in addition with their daily operations. As one leader reflected: *“We struggle with the data management needed to comply with our funding. We are too busy answering phones, providing care to improve our process.”*

### Facilitators

We inquired about facilitators to MOCU operations. Participants responded about facilitators broadly, describing facilitators to unit operations and patient success. We report on these facilitators below.

#### Patient and community-facing presence and engagement

MOCU leaders describe their placement in the community and their ability to bring services to individuals in need as the main reason for their success. Leaders described significant efforts in reaching out and engaging potential patients. Leaders described how their mobility as a van-based service helps reach people who otherwise wouldn’t have access to care, even in the hardest to reach areas. One leader described:

“*We can go down alleys, we can go under bridges, we can go into different encampments… with our huge [emergency medical treatment] bags.”*

Several of the MOCU leaders described travelling locally to find and provide services to individuals within the neighborhood. Leaders also described significant engagement and education efforts towards community members:

*“We then go out on foot into those neighborhoods, and we talk to local business owners. We want to know if they see a lot of overdoses, do they have Narcan? Do they have ‘Stop-The-Bleed Kits’? Do they have First Aid Kits? Are they comfortable responding to those things? If they aren't, can we train them?*”

About half of the MOCUs described working with community advisory boards to inform their location and services. As one leader described:

*“When it comes to making decisions for the program, we like to have their input also, because they are a part of the community where we provide service.”*

#### Interagency collaboration

MOCU leaders relied on interagency collaborations to improve care. In addition to connecting people to substance use and mental health treatment services, MOCU leaders reported connecting individuals to SSP, employment services, housing services, and medical services. Many of the MOCU leaders reported working with other MOCUs. MOCUs refer to each other to coordinate care, to refer patients in other geographic locations, and to ensure that services are not redundant. As one leader described:

*“We reach out to say, “Hey we’re going to be doing this …’ just so that we’re not duplicating services.”*

Several MOCUs collaborate with local police; at least one MOCU reported that the police refer individuals to their unit for care. Finally, many leaders stated that their collaboration with the city is critically important both for funding and because the city provides access to data about overdose hotspots.

#### Clinical teams

Unit leaders extol the value of their clinical staff. Leaders describe having staff familiar with the neighborhood greatly facilitates the unit’s operations:

“*We have a mobile research coordinator that’s been around, working here since we started, he is really familiar with the neighborhoods in Philadelphia and the gatekeepers in the community.”*

Outreach workers with experience in the neighborhood help choose the MOCU’s location and leverage social connections in the neighborhood for community acceptance and messaging the MOCU’s services to the population in need. Leaders also describe the importance of case managers on staff to address multiple social determinants of health. Case managers, outreach workers, and peers also provide warm handoffs to higher levels of care, give reminder calls for follow-up appointments, and empower clients about their patient rights. On some MOCUs, case managers accompany clients to their initial referral appointment to other substance use treatment services (e.g., IOP, OP or primary care-based treatment) to advocate and support them. As leaders describe all these functions “jumpstart” an individual’s capacity to engage in treatment on the MOCU and in the community. The staff are also skilled at adapting and “meeting people where they are.” Leaders described how staff conduct informal check-ins; some go on walks with clients or locate clients within the community to reduce stigma associated with being seen on the MOCU:

*“[Some clients] can be a little bit more private. For example, someone doesn't want to be seen necessarily at the Suboxone bus. I have seen in the past case managers and providers essentially walk the park with the participants. So it looks like a casual stroll, which has been really great*.”

## Discussion

This study of mobile OUD care service delivery models provides rich information about a novel service approach in response to the opioid and HIV epidemics. To our knowledge, this is the first study to describe and compare multiple providers of mobile OUD care delivery services in an urban setting. Consistent with previous research, we find that MOCUs are low barrier, humanistic, interdisciplinary modalities of care and present a promising opportunity to support individuals who have disengaged from traditional treatment systems [22, 34, 56–58]. Leaders described how their MOCUs embed themselves in neighborhoods of need and offer critical and often life-saving services to PWID, many of whom are unhoused. MOCUs also provide an important opportunity to deliver naloxone, case management, and HIV prevention services to this population. Leaders spoke of the tenacity, compassion, and flexibility of their clinical teams, crediting them with the success of their units.

We uncovered several challenges to mobile OUD care delivery. The major challenge shared by all programs was securing enough funding to sustain their operations. Most MOCUs reported being partially or completely supported by federal or city grants and many unit leaders said they would be unable to self-finance continued operations. All leaders reported an inability to pay staff adequately, which is concerning given staff turnover and potential for burnout among those who work in behavioral health services [59]. Permanent or other programmatic funding would facilitate MOCU operation and sustainability and ensure sufficient salaries for those who provide services. Mobile services may also require bundled or other alternative payment models to account for the medical and social complexity of the patients and reimburse the multidisciplinary services that typically occur on MOCUs. Although mobile care delivery models may be perceived as high intensity, they reach a high-risk population that is often poorly connected to other sources of care. Future work should focus on measuring the effectiveness of such models in improving outcomes, preventing acute and chronic complications of substance use, and reducing costs of care in high-need patients.

Leaders described their services as a “bridge” to connect disengaged and disenfranchised individuals back with the treatment system. Yet, leaders also described a substance use treatment system that either cannot or will not accommodate the type of individuals seen on the MOCU who are often unhoused, unemployed, and impoverished. This dialectic underscores a fundamental difference in the treatment approach of MOCUs (e.g., low-barrier, harm reduction) and some traditional treatment facilities that emphasize abstinence and promote punitive attendance policies. Leaders detail how their patients may not be comfortable at traditional treatment facilities or may struggle to adhere to strict requirements such as delayed treatment starts, mandatory attendance of counseling, or strict expectations of abstinence. These findings are consistent with a pilot study of a MOCU in Chicago, which found that a number of patients continued buprenorphine treatment on the MOCU instead of transitioning to a traditional health care facility [22].

While there is growing momentum for low-threshold treatment approaches (that prioritize medication access, flexibility, and harm reduction compared to more traditional treatment models [60, 61], treatment in brick-and-mortar facilities is still different from mobile, street-based models. Shifting incentives towards lower barrier, “medication first” practices may help bridge this divide, as well as offering more integrated services, including primary care, substance use treatment, HIV prevention and treatment and other services relevant to PWID. Finally, in addition to providing lifesaving services to individuals in need, MOCUs also provide a community service by providing education, distribution of harm reduction resources, and combatting stigma. However, MOCUs were not always welcomed by community members, and more work is needed to understand the most appropriate and effective ways to embed these services within communities.

Despite these differences, our data suggest that increased collaboration between MOCUs and brick-and-mortar sites is vital. Future research could explore “if” or how well individuals linked from mobile care are retained in community care as well as determinants of successful handoffs. Given that some individuals served by MOCU have no interest in returning to the traditional treatment setting, it may be beneficial for policy makers and funders to reconfigure MOCUs and their missions as permanent services instead of “gap-fillers.” MOCUs may be the only way to act on social determinants that stymie participation in traditional care.

Another notable finding is that buprenorphine was the primary MOUD provided. Almost all the MOCUs provide buprenorphine prescriptions; only one unit directly dispenses buprenorphine as part of a research study. None of the MOCUs were licensed opioid treatment programs and therefore could not provide methadone. There continue to be substantial regulatory, legal, and safety challenges to overcome for mobile providers to be able to provide MOUD on-site. In 2021, the Drug Enforcement Agency lifted a ban on mobile methadone provision, yet Pennsylvania law prohibited opioid treatment programs from having medication units that are geographically separated from the main site [62]. As of 2022, programs in Pennsylvania that wish to provide methadone mobile can request an exception that comes with stringent regulations [63]. The COVID-19 pandemic drove some regulatory easing for buprenorphine and methadone, and hopefully future legislation such as the Opioid Treatment Access Act and continued attention and advocacy will continue to uphold and extend this access [50, 51]. Future advocacy and research should focus on delivery models that provide access to methadone in similar low-barrier models.

## Limitations

This study has several limitations. The scope of this investigation is small and descriptive, with only seven MOCUs within a few neighborhoods in one city. Other cities or rural areas may face very different barriers and facilitators to mobile care. For example, recent survey studies have documented a lack of phone, Internet, and transportation access for individuals with SUD in rural areas of the United States, so MOCUs in these areas may have to rely more heavily on word-of-mouth and transport-to-treatment models to coordinate care [16, 64, 65]. Given the growth of mobile OUD care delivery models nationally, more broad scale evaluation of MOCUs is needed. Also, the service delivery landscape and the street drug supply is rapidly shifting and this study reports on the service delivery landscape at one moment in time [65]. A number of MOCU leaders described that they were in the process of implementing new services or expanding coverage to certain areas in the months following the interview.

## Conclusions

MOCUs are an emerging strategy to provide substance use treatment and other integrated services to PWID. MOCUs often take a harm reduction approach and show promise in overcoming many barriers to accessing care in more traditional models, but face shortfalls in funding and addressing the challenges of medically and socially complex PWID. Future work should focus on the role that MOCUs play in promoting access and effective care for this population.

### Supplementary Information

**Additional file 1. **Mobile OUD Care Unit Interview and Notes.

**Additional file 2. **Consolidated Criteria for Reporting Qualitative Research Checklist.

**Additional file 3.** Mobile OUD Care Unit Thematic Template.

## Data Availability

The datasets used and/or analyzed during the current study are available from the corresponding author on reasonable request.

## Abbreviations

OUD: Opioid Use Disorder
MOUD: Medication for opioid use disorder
PWID: Persons/people who inject drugs
MOCU: Mobile opioid use disorder care unit
RQA: Rapid Qualitative Analysis
SSP: Syringe service program
OPS: Overdose prevention site
