# Supplementary material for: Mobile service delivery in response to the opioid epidemic in Philadelphia
Source: Addict Sci Clin Pract. 2023 Nov 29;18:71. doi: 10.1186/s13722-023-00427-5 (PMC10687974; doi:10.1186/s13722-023-00427-5)
Supplement: Supplementary file 1 — Additional file 1. Mobile OUD Care Unit Interview and Notes. [file 13722_2023_427_MOESM1_ESM.pdf]

Mobile OUD Care Unit Interview and Notes

Date: __________ Unit: _____________ Interviewer Initial: ________

Coder 1: __________ Coder 2: ___________

Thank you so much for speaking with us today. We are researchers from the University of Pennsylvania's Center for Mental Health, and we're interested in the rise of non-traditional treatment modalities, especially mobile units in Philadelphia and across the country. We seek to systematically survey the landscape of mobile addiction treatment in the city, understand barriers and facilitators to your operation, and leverage with the existing mobile unit network to disseminate our findings and increase collaboration between the units.

This interview will be approximately 45-minutes and I'll first like to ask you about your unit, its staffing, operations, and patient capacity. Then, we will take some time to talk about the clinical approach, process, sustainment, and barriers that are unique to your unit.

This meeting will be recorded. We have approval from the Philadelphia IRB as a non-human subject's research study. Before I ask [Name] to begin recording, do you have any questions?

**About you**	
1. What is your title in your organization?	
**About the unit**	
1. Can you describe the physical space you work in? *Car, van, truck, trailer, etc.?*	1.
2. Do you know the make and model of your unit?	2.
3. How long has your unit been providing services?	3.
4. How many individuals staff your unit at a given time?	4.
5. What type of providers and staff do you have on your unit on a typical day? *Clinicians, peer specialists, induction specialists, etc.*	5.
**Capacity**	
1. Approximately how many clients do you have currently?	1.
2. What is your maximum caseload capacity?	2.
3. What is the average length of stay of people you enroll in the program?	3.
4. Approximately how many "touchpoints" do you have with a given client?	4.
5. How often do you schedule visits with your clients?	5.
6. Do you provide…	6a.
a. Medication induction…	6b.
b. Stabilization & referral…	6c.
7. Do you follow up with clients between visits?	7.
8. Do you perform drug screening on clients?	8.
**Location**	
1. Where are you typically located?	1.
2. How long do you stay in a given location?	2.
**Funding**	
1. How is your unit funded? (E.g. bill insurer, funding from a parent organization, federal funding, City of Philadelphia (e.g. DBH, DPH, CBH) funding, other grant funding)	

Mobile OUD Care Unit Interview and Notes

Rapid Qualitative Coding

Domain	Probes	Notes
Clinical Approach	1. How would you describe the therapeutic mission of your unit?	
Your Process	1. How do you pick a location?<br>2. What are the logistics involved with parking in an area on a given day?<br>3. Can you describe your intake/registration process?<br>4. What are the intake criteria?<br>5. What formal requirements does your program have to remain/continue in treatment?<br>6. Can you describe your process for follow-up appointments?<br>7. What are criteria for discharge from program?	1.<br>2.<br>3.<br>4.<br>5.<br>6.<br>7.
Sustainment	1. What services would you like to add/expand on?<br>2. What are the three services you would most like to add to your unit in the future?<br>3. What do you need to sustain your funding?	1.<br>2.<br>3.
Barriers	1. What is the most significant barrier preventing you from providing maximally effective service to your clients?<br>2. Are any other agencies interfering with your service?	1.<br>2.
Other	1. Are you familiar with any other organization providing mobile services?	

Mobile OUD Care Unit Interview and Notes

Services and Logistics Matrix

Do you provide…?		
**Medical Services**	**On-Site**	**Referral**
A. Pregnancy testing		
B. Viral hepatitis testing		
C. HIV testing		
D. Wound care		
**Medications**	**On-Site**	**Referral**
A. Naloxone distribution and education		
B. Dispense oral buprenorphine		
C. Provide prescription for buprenorphine		
D. Administer extended-release buprenorphine injection (e.g., Sublocade, Brixadi)		
E. Dispense methadone		
F. Administer extended-release naltrexone injection (Vivitrol)		
G. PrEP (pre-exposure prophylaxis)		
**Harm Reduction Services**	**On-Site**	**Referral**
A. Fentanyl test strip distribution		
B. Syringe exchange/return		
**Other Supports**	**On-Site**	**Referral**
A. Case management		
B. Peer specialist support		
C. Transportation assistance		
D. Assistance with housing		
E. Assistance with obtaining insurance/benefits		
F. Assistance with copays		
G. Basic supplies (food, water, toiletries, clothing)		
**Linkages and Referrals**	**Yes / No**	
A. Linkage to specialty substance use treatment outpatient programs		
B. Referrals to inpatient substance use treatment programs		
C. Referral to primary care (with or w/o buprenorphine)		
D. Referrals to primary care-based buprenorphine treatment		
E. Referrals to specialty mental health care		
