# Supplementary material for: Mobile service delivery in response to the opioid epidemic in Philadelphia
Source: Addict Sci Clin Pract. 2023 Nov 29;18:71. doi: 10.1186/s13722-023-00427-5 (PMC10687974; doi:10.1186/s13722-023-00427-5)
Supplement: Supplementary file 3 — Additional file 3. Mobile OUD Care Unit Thematic Template. [file 13722_2023_427_MOESM3_ESM.pdf]

Mobile OUD care unit themes

SHORT CODE: [unit name]	DATE:
PREPARED BY:	REVIEWED BY:
LOCATION:	
SUSTAINABILITY SOURCE(S):	
APPROACH FOR MOBILE DELIVERY	
FACILITATORS of IMPLEMENTATION	
BARRIERS of IMPLEMENTATION	
WHAT WOULD HELP	

Derived themes

-Data
-Interagency Collaboration
-Physical Space
-Location
-Patient and community-facing presence and engagement
-Staffing/ Clinical Teams
-Financing and funding model
-Treatment system
-Patient-facing Barriers
